# 

**Published:** 1924-10

**Authors:** 


### HOSPITAL and HEALTH REVIEW

*Established 1886* *No. 1879. Vol. LXXIII*

*New Series.* Vol. III. No. 37. *October,* 1924. Price Sixpence

CONTENTS

| | PAGE |
|---|---|
| A New Hospital System | 293 |
| Education in Health | 294 |
| Notes and Comments | 295 |
| The Final Distribution—Music and Medicine—Facts and Fiction about Opium—Factory Legislation—Work of the Asylum Board—Making Smallpox Easy—The Mentally Defective Child—The Teeth of the Army—A Land of Massacre—Paying Patients in America—Cleanliness in Schools—Health Talks at a County Fair—Beaver Coney—The Value of Home Nursing. | |
| Hospital Men of Mark: Col. A. C. Dawson, C.B.E., and Mr. Frank Inch | 299 |
| Scarcity of Hospital Almoners | 300 |
| Correspondence | 300 |
| The Problem of Cancer. By Dr. J. Hall-Edwards | 301 |
| Pensions for Nurses | 302 |
| The Majesty of Motherhood | 303 |
| The Lost Art of Hospital Ventilation | 304 |
| Tuberculosis in Nurses and Ward Maids | 305 |
| Smallpox in England and Wales | 305 |
| The Health Week Poster | 305 |
| An Ambitious Hospital Scheme | 306 |
| Population and Unemployment. By Col. Maurice, C.M.G. | 307 |
| Tests of Educable Capacity | 308 |
| Parochial Beds | 308 |
| Learning the Way of Health | 309 |
| Seamen and Tuberculosis | 310 |
| Health Work in the States | 312 |
| A Model Abattoir at Liverpool | 313 |
| A Hospital Chapel | 314 |
| Hospital Progress and Finance | 315 |
| Reviews of Books | 317 |
| Points from Hospital Reports | 321 |
| Echoes of the Month | 322 |
| Hospital and Health News | 323 |
| Recent Appointments | 325 |
| Answers to Correspondents | 325 |

